# Structural variation in the *IAMH2* 3′ UTR underlies hypocotyl elongation during light adaptation in *Arabidopsis thaliana*

**DOI:** 10.1111/tpj.71167

**Published:** 2026-10-07

**Authors:** Xuemeng Gao, Wenli Chang, Xuejing Liu, Yingjun Yao, Yan Song, Jin Yan, Kai Yang, Jianquan Liu, Huanhuan Liu

**Affiliations:** ^1^ Key Laboratory for Bio‐resources and Eco‐environment & Sichuan Zoige Alpine Wetland Ecosystem National Observation and Research Station, College of Life Sciences Sichuan University Chengdu 610065 China; ^2^ College of Horticulture and Gardening Yangtze University Jingzhou 434025 Hubei China; ^3^ Chengdu Botanical Garden Chengdu 610000 China; ^4^ State Key Laboratory of Herbage Improvement and Grassland Agro‐Ecosystems and College of Ecology Lanzhou University Lanzhou 730000 China

**Keywords:** hypocotyl elongation, *IAMH2*, 3′ UTR structural variation, posttranscriptional regulation, *Arabidopsis thaliana*, low‐light adaptation

## Abstract

Plant hypocotyls are central to seedling establishment in variable light environments. Although core photoreceptor pathways regulating hypocotyl elongation are well defined, the molecular basis of natural variation in this trait—particularly through posttranscriptional regulation—remains poorly understood. Here, we report that a high‐altitude, high‐light‐adapted Arabidopsis ecotype from Tibet exhibits longer hypocotyls under low light compared to a low‐altitude ecotype from Sichuan. Using recombinant inbred lines combined with fine‐mapping and near‐isogenic line validation, we identified the major locus *LA1* (*Light Adaptation 1*) and pinpointed *IAMH2* as the causal gene. Full‐length transcriptomics revealed a natural structural variant in the *IAMH2* 3′ UTR of high‐altitude ecotypes that alters its length and disrupts a poly(U) motif. We further show that in low‐altitude ecotypes, the RNA‐binding proteins C3H14/C3H15 directly bind this poly(U)‐rich region to posttranscriptionally downregulate *IAMH2* mRNA levels, thereby attenuating *IAMH2*‐dependent IAA accumulation and hypocotyl elongation. Analysis of 38 globally representative ecotype genomes confirmed that *IAMH2* 3′ UTR variation correlates with light intensity and elevation, supporting its role in environmental adaptation. Collectively, our work reveals a previously unknown C3H14/C3H15–*IAMH2* posttranscriptional module that dynamically adjusts hypocotyl elongation in response to light, providing new insights into the plasticity and adaptive evolution of seedling photomorphogenesis.

## Significance Statement

Noncoding genetic variation is an important but underexplored source of phenotypic diversity. This study shows that a 269‐bp structural variant in the *IAMH2* 3′ UTR enables high‐altitude Arabidopsis ecotypes to escape C3H14/C3H15‐mediated posttranscriptional repression, leading to elevated *IAMH2* expression and enhanced hypocotyl elongation under low light. The findings establish a mechanistic link between a specific structural variant and an ecologically relevant growth trait.

## INTRODUCTION

The hypocotyl serves as a pivotal environmental sensing and signal‐integrating organ in plants, capable of synergistically interpreting diverse external cues such as light, temperature, and soil mechanical stress (Krahmer & Fankhauser, 2024). Light signals are transduced into intracellular responses via specific photoreceptors, including phytochromes, cryptochromes, and UVR8, thereby regulating multiple developmental phases from seed germination and seedling emergence to floral transition (Chen et al., 2004; Paik & Huq, 2019; Podolec et al., 2021). By integrating light information into endogenous genetic networks, plants dynamically reprogram their developmental trajectories (Yadav et al., 2020). The elongation capacity of the Arabidopsis hypocotyl is crucial for adaptation to variable light environments. Under darkness or low light, seedlings undergo rapid hypocotyl elongation to reach the soil surface, driven by the alleviation of light‐imposed growth suppression (e.g., via gibberellin‐promoted degradation of DELLA proteins) (Bawa et al., 2020; Feng et al., 2008). Upon perceiving sufficient light, phytochrome/cryptochrome‐mediated signaling pathways are rapidly activated, suppressing cell elongation and promoting photomorphogenesis (Alabadí et al., 2004; de Wit et al., 2016). This developmental switch relies on extensive crosstalk between light signaling and phytohormone networks, with auxin (indole‐3‐acetic acid, IAA) acting as a central metabolic and morphogenetic integrator (Oh et al., 2014; Casanova‐Sáez et al., 2021). Studies demonstrate that auxin regulation of hypocotyl elongation exhibits marked dose‐ and light‐dependence: light conditions systematically alter the hypocotyl response curve to auxin concentration, thereby enhancing seedling adaptability to auxin‐mediated growth inhibition and enabling precise acclimation to the ambient light environment (Wang et al., 2025).

The spatial concentration gradient of IAA is precisely maintained by a dynamic equilibrium among its biosynthesis, polar transport, and reversible inactivation (Ljung, 2013; Casanova‐Sáez et al., 2021). Among the four known tryptophan‐dependent IAA biosynthetic pathways, the indole‐3‐pyruvate (IPyA) pathway is regarded as the primary source of IAA in plants (Casanova‐Sáez et al., 2021). Concurrently, the role of the indole‐3‐acetamide (IAM) pathway has become increasingly defined. Initially characterized in plant‐pathogenic bacteria, this pathway has now been validated across diverse plant species (Mano et al., 2010; Mano & Nemoto, 2012). The conversion of IAM to IAA depends on specific IAM hydrolases. In *Arabidopsis thaliana*, two tandem IAM hydrolase genes—*IAMH1* and *IAMH2*—have been reported to catalyze the terminal step in the IAM‐to‐IAA conversion reaction, and the *iamh1 iamh2* double mutant exhibits insensitivity to exogenous IAM and suppresses the high‐auxin phenotype induced by overexpression of the bacterial IAM synthase gene *iaaM* (Gao et al., 2020).

Alternative splicing and differential mRNA stability—often determined by cis‐regulatory elements within the 3′ untranslated region (3′ UTR)—represent key posttranscriptional layers in the genetic regulation of quantitative traits (Litterman et al., 2019; Mayr, 2017, 2019). For instance, under high‐light stress in cucumber seedlings, the preferential production and accumulation of a nonfunctional splice variant, *CsGA2ox8.2*, sustains rapid growth by attenuating the degradation of active gibberellins (Liu et al., 2021). In poplar, the RNA‐binding protein PdGRP1 precisely modulates brassinosteroid‐mediated secondary growth by binding to GU‐rich elements in the 3′ UTR of *PdCPD1* transcripts to promote their decay (Wang et al., 2023). Similarly, in *A. thaliana*, tandem CCCH‐type zinc finger proteins C3H14 and C3H15 instigate the decay of target mRNAs by recognizing poly(U) and AU‐rich elements (AREs) in their 3′ UTRs, thereby positively regulating secondary wall formation and anther development (Chai et al., 2015; Kim et al., 2014); furthermore, C3H15 also transcriptionally represses *BZR1* and *BES1* to negatively regulate cell elongation (Chai et al., 2022). Notably, within the ethylene signaling pathway, the 3′ UTR of *EBF1/2* mRNA functions as a molecular relay; its unique poly(U) motif, recognized by the EIN2 protein, directs the transcript into cytoplasmic processing bodies (P‐bodies) for translational repression (Li et al., 2015). These examples collectively underscore the central role of 3′ UTRs in integrating environmental signals with developmental programs. However, whether analogous posttranscriptional regulatory mechanisms are involved in fine‐tuning hypocotyl elongation and thereby influencing plant adaptability to light environments remains unclear.

In this study, we found that under low‐light conditions, the hypocotyl of the *A. thaliana* Tibet ecotype (Tib) from high‐light habitat was significantly longer than that of the Sichuan ecotype (Sic) from low‐light habitat. To identify the key genetic locus regulating hypocotyl elongation in response to light intensity, designated as *Light Adaptation 1* (*LA1*), we conducted a structural variant genome‐wide association study (SV‐GWAS) using recombinant inbred lines (RILs) derived from a cross between the Sic and Tib ecotypes (Lou et al., 2022). This study identifies *IAMH2* (*LA1*) as the causal gene underlying natural variation in hypocotyl elongation. In the Tib ecotype, the *IAMH2* 3′ UTR carries an additional 269‐bp sequence that disrupts a poly(U) loop. This structural alteration disrupts mRNA decay mediated by C3H14 and C3H15, ultimately promoting hypocotyl elongation, at least in part through enhanced IAA accumulation. We thus link a noncoding structural variant to adaptive growth through posttranscriptional regulation, revealing a previously uncharacterized mechanism in the IAM pathway that elucidates how plants modulate organ growth to adapt to varying light conditions.

## RESULTS

### GWAS‐based fine mapping identifies *IAMH2* as a regulator of hypocotyl elongation under low light

High altitude environments are characterized by elevated light intensities, which significantly influence plant developmental adaptations (Zhang et al., 2021). *A*. *thaliana* ecotypes originating from regions with high (Tib) and low (Sic) light intensities (Figure S1A, B) exhibit distinct morphological adaptations, particularly in hypocotyl elongation responses to varying light conditions. Phenotypic analysis revealed that the Tib ecotype exhibits a significantly more sensitive hypocotyl elongation response to decreasing light intensity than the Sic ecotype (Figure 1A, B). Under standard laboratory growth light (normal condition), the hypocotyl of the Tib ecotype was approximately 1 mm longer than that of the Sic ecotype. This difference increased to over 3.5 mm under low light, demonstrating a significant divergence in hypocotyl elongation response under low light between these two ecotypes (Figure 1A, B).

**Figure 1:**
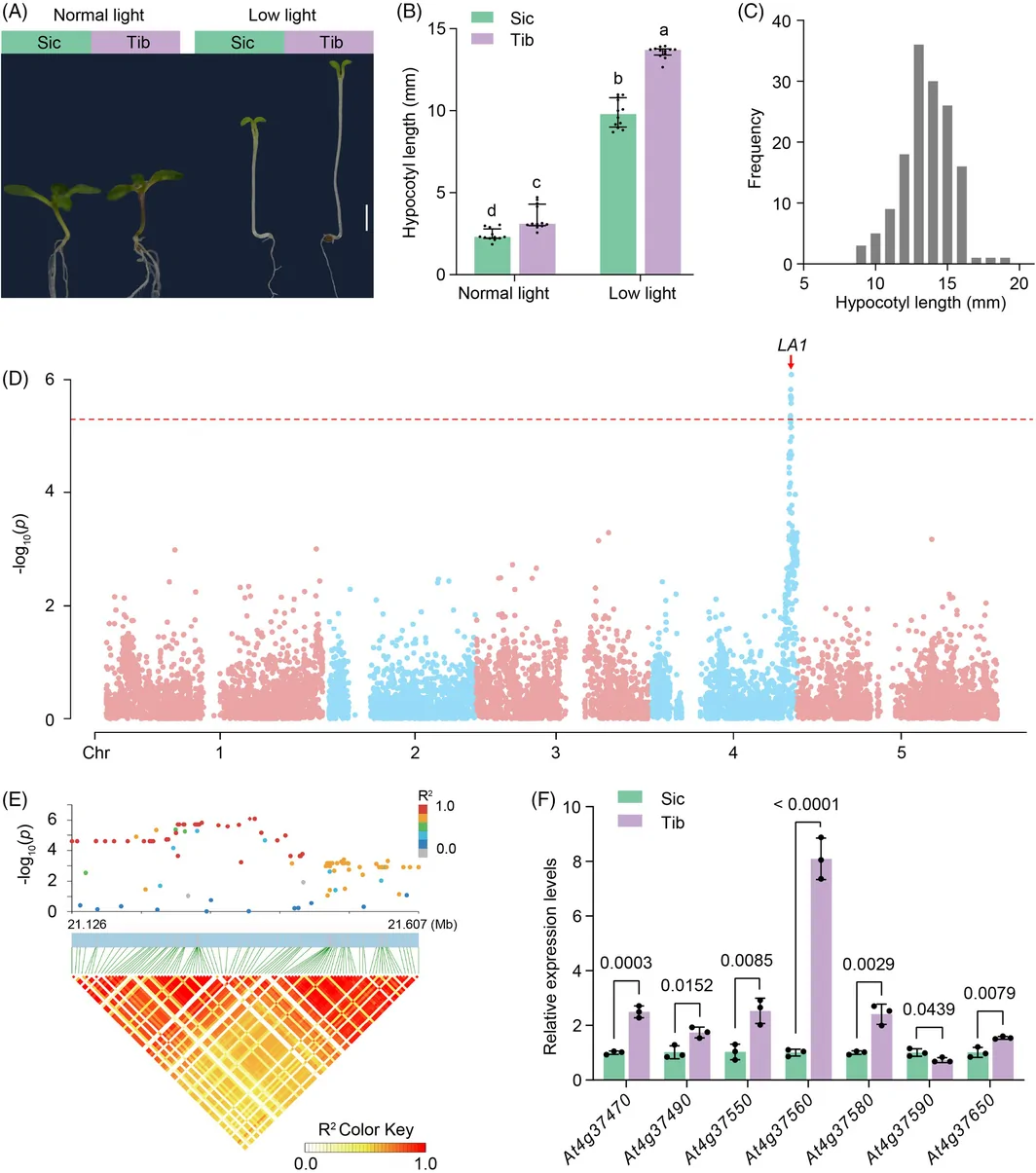
Genome‐wide association mapping of hypocotyl length in *Arabidopsis thaliana*. (A) Representative images of 9‐day‐old Sichuan (Sic) and Tibet (Tib) seedlings grown under normal and low light. Scale bar, 2 mm. (B) Quantification of hypocotyl lengths corresponding to (A). Data are mean ± SD (n = 21 and 26 seedlings, respectively), different letters denote statistically significant differences (*P* < 0.05, one‐way ANOVA with Tukey's multiple‐comparisons test). (C) Frequency distribution of hypocotyl length in the RILs under low light. (D) Manhattan plot from the structural‐variant GWAS (SV‐GWAS) of hypocotyl length under low light. The dashed red line indicates the 5% Bonferroni‐corrected genome‐wide significance threshold. (E) Regional association signals and linkage‐disequilibrium heatmap surrounding the peak locus on chromosome 4 (21.126–21.607 Mb). (F) RT‐qPCR analysis of seven candidate genes in manually dissected hypocotyls from 9‐day‐old Sic and Tib seedlings grown under low light (7 μmol m^−2^ s^−1^). Data are mean ± SD of three biological replicates; *P* values from unpaired two‐tailed Student's *t*‐test.

To identify the major locus *LA1* underlying hypocotyl elongation response between these two ecotypes, we conducted a GWAS analysis. We first quantified hypocotyl length using 146 F_7_ recombinant inbred lines derived from a cross between the Sic and Tib ecotypes (Lou et al., 2022) under low‐light conditions. The trait showed a continuous, approximately normal distribution in the RIL population (Figure 1C), suggesting a polygenic architecture. Given increasing evidence that structural variations (SVs) can explain more phenotypic variance than SNPs (He et al., 2025; Kang et al., 2023), we conducted an SV‐GWAS by combining hypocotyl length data under low light with whole‐genome resequencing. The analysis yielded a robust association signal in the distal region of chromosome 4, which fine‐mapped to a major genetic locus *LA1* (Figure 1D). Using structural variant (SV) markers, we assessed linkage disequilibrium (LD) patterns across a ∼481 kb region flanking the peak, which revealed clearly demarcated LD blocks in this genomic interval (Figure 1E). Within this defined genomic interval, we systematically screened candidate genes with known or predicted functions in plant hormone signaling or growth and development pathways, ultimately identifying seven high‐confidence candidate genes (Figure 1F; Data S1). To evaluate their potential involvement in hypocotyl elongation divergence between Tib and Sic, we employed RT‐qPCR to analyze their transcript abundance in manually dissected hypocotyls from 9‐day‐old seedlings grown under low‐light conditions. Among these candidates, *At4g37560* (*IAMH2*) exhibited the most pronounced elevation in expression levels (>eightfold higher) in the Tib ecotype compared to the Sic ecotype (Figure 1F), suggesting a putative role in hypocotyl elongation divergence under low‐light conditions. This difference was also significant under normal light (Figure S1C).

To further identify the genetic determinants underlying natural variation in hypocotyl photosensitivity, we integrated traditional linkage mapping with a breeding strategy by crossing the Tib and Sic ecotypes, followed by backcrossing with the Tib ecotype to generate chromosome segment substitution lines (CSSLs; Figure S2). Analysis of PCR markers across the BC_5_F_5_ CSSLs populations localized a hypocotyl elongation‐associated locus to a ∼13 kb genomic region containing only five genes (Figure 2A). RT–qPCR and phenotypic analyses revealed that only the expression level of *At4g37560* was strongly correlated with longer hypocotyls under low‐light conditions in the CSSLs (Figure 2B–D). Furthermore, structural variation was uniquely detected in *At4g37560* (*IAMH2*), suggesting that allelic variation in this gene may underlie the differential hypocotyl elongation response between Tib and Sic ecotypes (Figure 2E; Figures S3 and S4).

**Figure 2:**
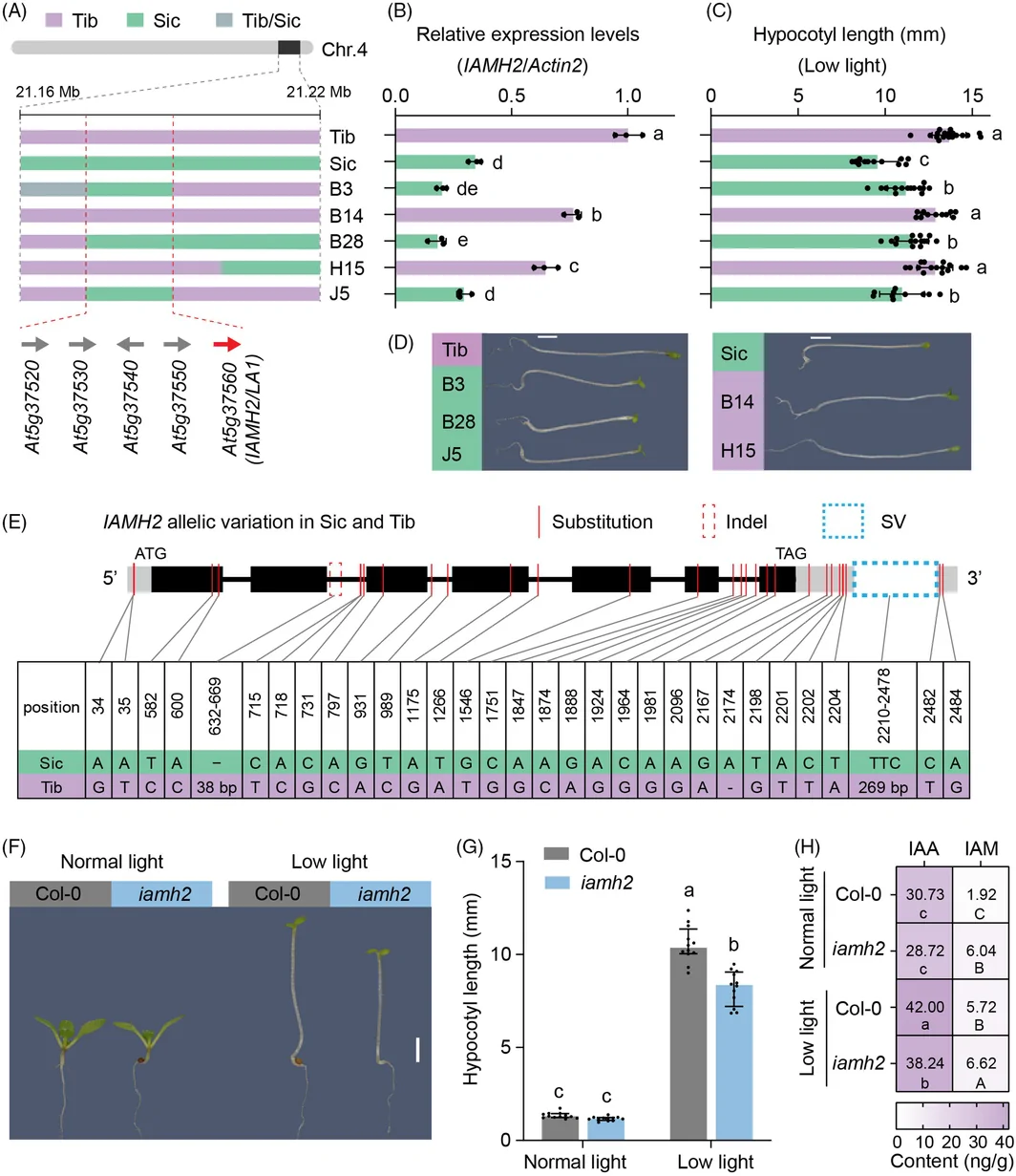
Identification of *IAMH2* as a regulator of hypocotyl elongation under low light. (A) Fine‐mapping of the major hypocotyl‐length locus on the Tibet ecotype genomic background. (B) RT‐qPCR analysis of *IAMH2* expression in manually dissected hypocotyls from 9‐day‐old seedlings of the five NILs and the parents grown under low light (7 μmol m^−2^ s^−1^). (C) Quantification of hypocotyl lengths corresponding to (D). (D) Representative images of 9‐day‐old NIL and parental seedlings under low light. Scale bar, 2 mm. (E) Gene structure and allelic variations of *IAMH2* between the Sichuan and Tibet ecotypes. (F) Representative images of 9‐day‐old Col‐0 and *iamh2* mutant seedlings under normal and low light. Scale bar, 2 mm. (G) Hypocotyl length quantification for the conditions shown in (F). (H) Quantification of IAA and IAM levels in manually dissected hypocotyls from 9‐day‐old Col‐0 and *iamh2* seedlings grown under normal and low light. In (b), data are mean ± SD (n = 3 biological replicates). In (C) and (G), data are mean ± SD (n = 18/12/14/11/15/15/10 and 12 seedlings, respectively). In (h), data are mean ± SD (n = 3 biological replicates). Different letters in (B), (C), and (G) denote statistically significant differences (*P* < 0.05, one‐way ANOVA with Tukey's multiple‐comparisons test). In (H), lowercase letters denote significant differences for IAA, and uppercase letters denote significant differences for IAM (*P* < 0.05, one‐way ANOVA with Tukey's multiple‐comparisons test).

*At4g37560* encodes an indole‐3‐acetamide hydrolase (IAMH2) that catalyzes the hydrolysis of IAM to IAA (Gao et al., 2020). To establish a direct functional link, we analyzed the *iamh2* T‐DNA insertion mutant SALK_208590C, obtained from NASC. The T‐DNA insertion site and the loss of full‐length *IAMH2* transcript in this allele were confirmed (Figure S5). Under low light, the *iamh2* mutant exhibited a significantly shorter hypocotyl compared to the Col‐0 wild type (Figure 2F, G). This genetic evidence confirms that *IAMH2* is a positive regulator of hypocotyl elongation, likely by modulating auxin homeostasis in response to light cues. To determine whether the shortened hypocotyl of the *iamh2* mutant is accompanied by altered auxin metabolism, we measured IAA and IAM levels specifically in manually dissected hypocotyls. Under low‐light conditions, IAA levels were significantly lower in *iamh2* than in Col‐0, whereas under normal light, the reduction was not statistically significant (Figure 2H). IAM levels were significantly elevated in the mutant under normal light (Figure 2H), consistent with reduced IAM hydrolysis upon loss of *IAMH2*. These data indicate that *IAMH2* contributes to the hypocotyl IAA pool, particularly under low light.

### Structural variant in 3′ UTR ablates a conserved RNA motif

To elucidate the allelic variations in *IAMH2* underlying functional divergence between the Sic and Tib ecotypes, we analyzed its gene sequences and compared the deduced protein sequences and structures. While three amino acid substitutions were found in the coding sequence, none were located within functional domains or predicted to affect protein structure (Figure S6). Instead, we identified a structural variation in the 3′ UTR, with the Tib allele carrying an additional 269‐bp sequence relative to the Sic allele (Figure 2E).

To characterize the 3′ UTR polymorphism, we performed full‐length transcriptome analysis using PacBio long‐read sequencing and Illumina PE150 paired‐end sequencing (Figure 3A, B). Contrary to the expectation that an insertion would lengthen the transcript, comparative analysis between ecotypes revealed that the 269‐bp structural variant in the Tib allele ultimately resulted in a shorter mature 3′ UTR—specifically, a 34‐bp reduction compared to the Sic allele (Figure 3C). This indicated that the variant alters the pattern of transcript termination or processing, generating distinct *IAMH2* ^*Tib*^ and *IAMH2* ^*Sic*^ isoforms with divergent 3′ UTR sequences and lengths. We next analyzed the RNA secondary structure to assess the functional impact of the sequence divergence. Our predictions revealed a critical architectural distinction: the *IAMH2* ^*Tib*^ 3′ UTR lacks a characteristic single‐stranded loop containing a conserved poly(U) motif, which is present in both the *IAMH2* ^*Sic*^ and *IAMH2* ^*Col‐0*^ alleles (Figure 3D–F). The absence of this motif in the Tib allele indicates the loss of a key cis‐regulatory element, providing a structural basis for the possible differential posttranscriptional regulation of *IAMH2* across ecotypes.

**Figure 3:**
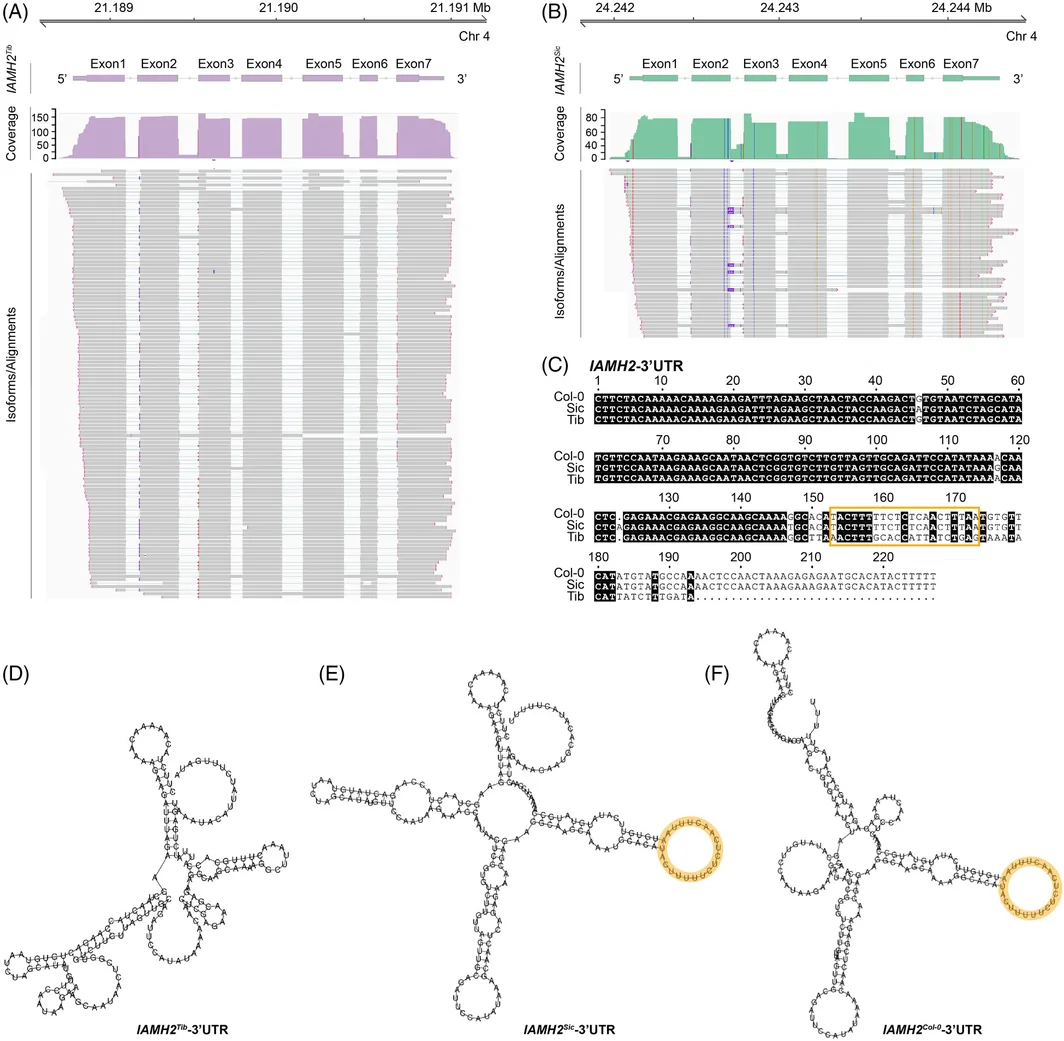
A 3′ UTR structural variant underlies differential *IAMH2* transcript architecture. (A, B) Full‐length transcriptome profiles (PacBio long‐read RNA‐seq) of *IAMH2* in the Tib (A) and Sic (B) ecotypes. Tracks display the gene model, read‐coverage depth, consensus isoform structures, and representative read alignments. (C) Sequence alignment of the IAMH2 3′ UTR from Col‐0, Sic, and Tib alleles. (D–F) Predicted secondary structures of the 3′ UTR for Col‐0 (D), Sic (E), and Tib (F) alleles, generated with RNAfold. A conserved single‐stranded loop motif critical for RNA–protein interaction is highlighted in orange.

### C3H14 and C3H15 posttranscriptionally regulate *IAMH2* expression via 3’ UTR binding

Given the role of the 3′ UTR in posttranscriptional control, we hypothesized that the poly(U) motif is targeted by specific trans‐acting regulators. C3H14 and C3H15, CCCH‐type RNA‐binding proteins previously reported to bind the poly(U) motif (Chai et al., 2015) and suppress cell elongation (Chai et al., 2022), emerged as strong candidate regulators. To investigate whether these repressors are themselves regulated by light, we compared their transcript levels in Col‐0 hypocotyls under normal and low light. Both genes were expressed at significantly higher levels under normal light than under low light (Figure 4A), suggesting that *C3H14* and *C3H15* are transcriptionally responsive to light conditions. Consistent with their proposed role as negative regulators, overexpression of either protein in the Col‐0 background resulted in significantly shorter hypocotyls (Figure 4B, C).

**Figure 4:**
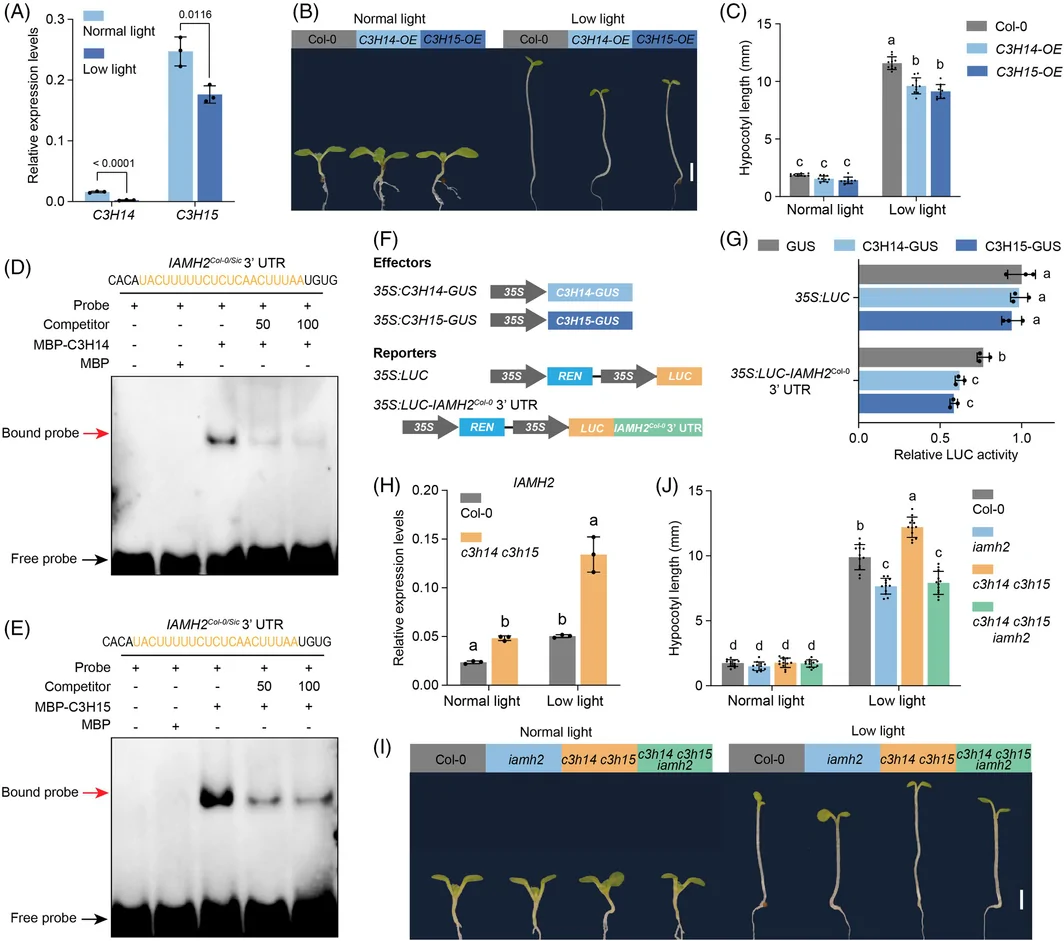
C3H14 and C3H15 negatively regulate *IAMH2* abundance through direct interaction with its 3′ UTR. (A) RT‐qPCR analysis of *C3H14* and *C3H15* expression in manually dissected hypocotyls from 9‐day‐old Col‐0 seedlings under normal or low light. Data are mean ± SD (n = 3 biological replicates); significance evaluated by unpaired two‐tailed Student's *t*‐test. (B) Representative images of 9‐day‐old Col‐0, *C3H14‐OE*, and *C3H15‐OE* seedlings under normal light or low light. Scale bar, 2 mm. (C) Quantification of hypocotyl lengths corresponding to (b). Data are mean ± SD (n = 10 seedlings). (D, E) RNA‐EMSA demonstrating specific binding of recombinant MBP‐tagged C3H14 (D) and C3H15 (E) to the *IAMH2* 3′ UTR probe; MBP alone served as a negative control. (F) Schematic of the effector and reporter constructs used in transient expression assays. (G) Results of dual‐luciferase reporter assays evaluating the effect of C3H14 or C3H15 co‐expression on *IAMH2* 3′ UTR‐driven reporter activity. Data are mean ± SD (n = 3 biological replicates). (H) RT‐qPCR analysis of *IAMH2* expression in manually dissected hypocotyls from 9‐day‐old Col‐0 and *c3h14 c3h15* seedlings under normal or low light. Data are mean ± SD (n = 3 biological replicates). (I) Representative images of 9‐day‐old Col‐0, *iamh2*, *c3h14 c3h15, and iamh2 c3h14 c3h15* seedlings under normal and low light. Scale bar, 2 mm. (J) Quantification of hypocotyl lengths in (i). Data are mean ± SD (n = 12 seedlings). In (C), (G), (H), and (J), different letters denote statistically significant differences (*P* < 0.05, one‐way ANOVA with Tukey's multiple‐comparisons test).

To determine whether C3H14 and C3H15 directly interact with the poly(U) motif within the 3′ UTR of *IAMH2*, we performed RNA electrophoretic mobility shift assays (RNA‐EMSAs). The results confirmed specific binding of both C3H14 and C3H15 proteins to the *IAMH2* RNA probe of Col‐0/Sic (Figure 4D, E). We next assessed the functional consequence of this interaction using a dual‐luciferase reporter system. Effector plasmids expressing C3H14 or C3H15 were co‐transfected with a reporter in which the Col‐0/Sic *IAMH2* 3′ UTR was fused to a 35S‐driven *LUC* gene (Figure 4F). The *IAMH2* 3′ UTR alone substantially reduced basal LUC activity relative to a UTR‐less control. Co‐expression of C3H14 or C3H15 further and significantly repressed reporter expression (Figure 4G). These results demonstrate that the *IAMH2* 3′ UTR contains cis‐acting repressive elements and that C3H14/C3H15 act as trans‐regulators that bind directly to this region to enhance posttranscriptional repression.

To confirm this regulatory relationship in vivo, we examined *IAMH2* transcript levels in the *c3h14 c3h15* double mutant. *IAMH2* expression was significantly elevated in the mutant compared with Col‐0 under both normal and low light (Figure 4H), demonstrating that C3H14 and C3H15 repress endogenous *IAMH2* expression. To further determine whether C3H14 and C3H15 regulate hypocotyl elongation through *IAMH2*, we generated the *iamh2 c3h14 c3h15* triple mutant. Under low light, the triple mutant exhibited hypocotyl lengths comparable to those of the *iamh2* single mutant (Figure 4I, J), indicating that *IAMH2* is epistatic to *C3H14* and *C3H15*. Under normal light, hypocotyl lengths of *iamh2*, *c3h14 c3h15*, and *iamh2 c3h14 c3h15* did not differ significantly from those of Col‐0 (Figure 4I, J), indicating that this regulatory module exerts its phenotypic effect specifically under low light. Collectively, these *in vivo* genetic data establish that *C3H14* and *C3H15* regulate hypocotyl elongation under low light conditions via *IAMH2*.

### Shortened 3′ UTR prevents C3H14/C3H15 binding and maintains *IAMH2* levels in Tib

We then asked whether the structural variation of *IAMH2* between the Sic and Tib alleles underlies their differential regulation. To test this, we generated 35S promoter‐driven LUC reporter constructs containing either the *IAMH2* ^*Sic*^‐3′ UTR or *IAMH2* ^*Tib*^‐3′ UTR sequences (Figure 5A). Dual‐luciferase reporter assays demonstrated that the *IAMH2* ^*Sic*^‐3′ UTR significantly reduced LUC activity, whereas the *IAMH2* ^*Tib*^‐3′ UTR had no effect on LUC expression (Figure 5B). When co‐expressed with C3H14 and C3H15 effectors, the LUC‐*IAMH2* ^*Sic*^‐3′ UTR reporter exhibited significantly reduced LUC expression compared to the LUC‐*IAMH2* ^*Tib*^‐3′ UTR reporter (Figure 5B). Consistent with these findings, RNA‐EMSAs revealed that C3H14 and C3H15 failed to bind to the *IAMH2* ^*Tib*^ RNA probe but specifically recognized and bound to the *IAMH2* ^*Sic*^ RNA probe (Figures 4C, D and 5C). Collectively, these data establish a direct link from DNA sequence variation to mRNA structural alteration to regulatory outcome: the additional 269‐bp sequence in the Tib 3′ UTR shortens the 3′ UTR and abolishes a conserved poly(U)/ARE motif. This structural alteration impedes recognition and binding by the repressors C3H14 and C3H15, thereby abolishing posttranscriptional repression, leading to elevated *IAMH2* expression (Figure 5B) and ultimately leading to enhanced hypocotyl elongation in Tib.

**Figure 5:**
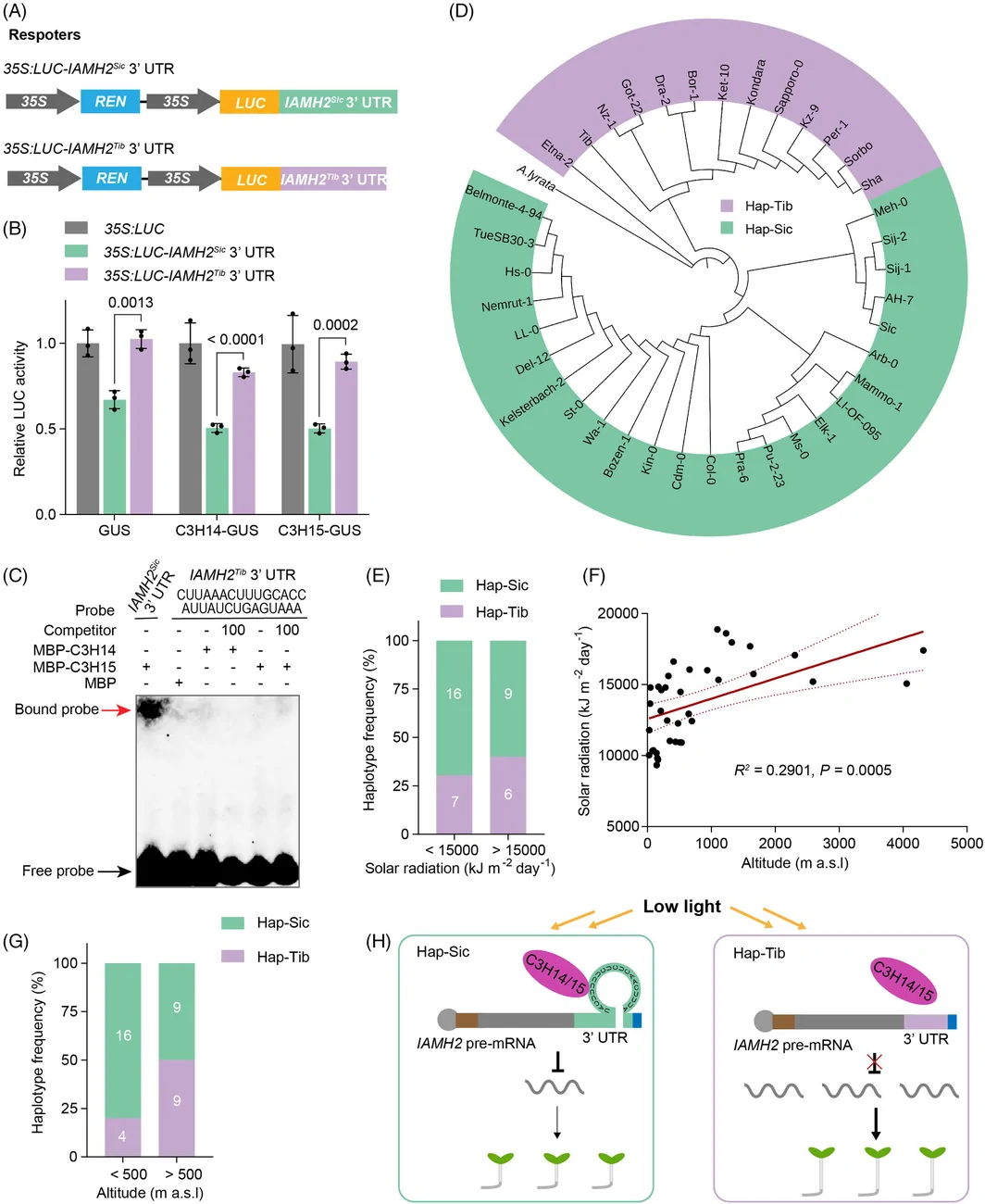
The Tib allele of *IAMH2* escapes posttranscriptional repression and is associated with high‐light adaptation. (A) Schematic of the dual‐luciferase reporter constructs used for transient expression assays. (B) Dual‐luciferase assays were conducted to assess the 3′ UTR activity of *IAMH2* ^*Sic*^ and *IAMH2* ^*Tib*^ in response to modulation by C3H14 and C3H15. Data are mean ± SD (n = 3 biological replicates); *P* value from unpaired two‐tailed Student's t‐test. (C) RNA‐EMSA demonstrating specific binding of recombinant C3H14 and C3H15 to the *IAMH2* ^*Tib*^ 3′UTR probe. (D) Maximum‐likelihood phylogeny of 38 natural *A. thaliana* ecotypes based on all *IAMH2* mRNA polymorphisms. Ecotypes carrying the Hap‐Tib are highlighted in green; Hap‐Sic ecotypes are highlighted in purple. (E) Haplotype frequency of Hap‐Tib and Hap‐Sic among ecotypes from regions with different solar‐radiation levels. (F) Correlation between solar radiation and altitude across the 38 ecotype sites. (G) Haplotype frequency of Hap‐Sic and Hap‐Tib among ecotypes in different altitude regions. (H) Working model: The natural splice variant in the *IAMH2* 3′ UTR alters the RNA's affinity for C3H14 and C3H15, thereby fine‐tuning *IAMH2* transcript levels and hypocotyl elongation under low‐light conditions. In Hap‐Tib, the absence of the poly(U) loop prevents recognition and repression by C3H14/C3H15, leading to enhanced hypocotyl elongation, whereas Hap‐Sic remains susceptible to repression.

To directly test whether the 3′ UTR structural variant is sufficient to confer differential hypocotyl elongation, we generated transgenic Col‐0 lines expressing *IAMH2* CDS–3′ UTR^Sic^ or CDS–3′ UTR^Tib^ under the *CaMV 35S* promoter. Because both constructs share the identical Col‐0 CDS, any phenotypic difference between the transgenic lines can be attributed solely to the 3′ UTR. Under low light, seedlings carrying CDS–3′ UTR^Tib^ developed significantly longer hypocotyls than those carrying CDS–3′ UTR^Sic^ (Figure S7A, B). RT‐qPCR confirmed that the CDS–3′ UTR^Tib^ lines accumulated correspondingly higher *IAMH2* transcript levels (Figure S7C). These results provide in vivo evidence that the 3′ UTR variant is a functional causal element underlying differential *IAMH2* expression and hypocotyl elongation.

### The structural variation of *IAMH2* 3′ UTR correlates with light adaptation in *A. thaliana*

To investigate the natural variation in the *IAMH2* 3′ UTR across globally distributed *A. thaliana* ecotypes, we analyzed orthologous sequences from 38 geographically diverse, representative *A. thaliana* ecotypes for which high‐quality genome assemblies were available, along with one *Arabidopsis lyrata* (*A. lyrata*) outgroup (Data S2 and S2). Phylogenetic reconstruction delineated two distinct monophyletic clades: one clade comprised 13 Tib‐haplotypes (Hap‐Tib) carrying the 3′ UTR structural variant, while the other contained 25 Sic‐haplotypes (Hap‐Sic) lacking this variant (Figure 5D). This phylogenetic pattern suggests that the variant likely represents an ancestral polymorphism that has been maintained and distributed across multiple ecotypic lineages, rather than arising from recurrent independent mutations.

To assess whether haplotype distribution reflects environmental adaptation, we evaluated its correlation with key habitat parameters. The Hap‐Tib was significantly enriched in regions with higher solar radiation (Figure 5E), a direct proxy for ambient light intensity. Importantly, solar radiation was tightly correlated with elevation across all sampled sites (Figure 5F). In accordance with this environmental association, Hap‐Tib was also predominant in higher altitude habitats relative to Hap‐Sic (Figure 5G). Taken together, these findings indicate that the *IAMH2* 3′ UTR variant exhibits a geographic distribution associated with high‐altitude, high‐light environments, representing a discernible signature of local adaptation to these conditions.

## DISCUSSION

Rapid hypocotyl elongation is critical for seedling emergence and the transition to autotrophic growth (Krahmer & Fankhauser, 2024). Through comparative analysis of *A. thaliana* ecotypes from high‐light (Tib) and low‐light (Sic) habitats, this work delineates a complete regulatory pathway from natural variation to adaptive phenotype (Figure S1; Figure 1A, B). We demonstrate that a 269‐bp structural variant in the 3′ UTR of *IAMH2* underlies the divergence in hypocotyl elongation response (Figure 5H). This variant shortens the transcript and, critically, ablates a conserved poly(U)‐containing loop that serves as a *cis*‐regulatory motif for the RNA‐binding proteins C3H14 and C3H15. Consequently, the Tib allele escapes posttranscriptional repression, leading to enhanced *IAMH2* mRNA level and driving exaggerated hypocotyl elongation under low light (Figure 5H). Thus, our study establishes a direct link between noncoding allelic variation and a key adaptive trait, revealing a previously uncharacterized posttranscriptional mechanism that fine‐tunes organ growth to ambient light intensity.

The biosynthesis of IAA involves multiple pathways (Mashiguchi et al., 2011). Although IAMH1 and IAMH2 catalyze the conversion of IAM to IAA (Gao et al., 2020), recent studies have questioned whether this pathway contributes to auxin biosynthesis under standard conditions (Moya‐Cuevas et al., 2026). IAA levels were significantly reduced in *iamh2* hypocotyls under low light, whereas the reduction under normal light was not significant, and IAM accumulated in the mutant under normal light (Figure 2H). We therefore propose that the IAM pathway is not essential for basal auxin homeostasis, but is conditionally recruited under low‐light stress to provide local IAA for adaptive growth. This model reconciles the lack of a strong IAA phenotype in *iamh1 iamh2* mutants under normal growth (Gao et al., 2020) with the pronounced hypocotyl divergence between ecotypes specifically under low light. Tib and Sic differed in hypocotyl length even under normal light, whereas *iamh2* did not differ from Col‐0, suggesting that natural variation in *IAMH2* expression can modulate growth, while complete loss of *IAMH2* is buffered under normal light. Notably, Sic and Tib inhabit brighter environments than our experimental normal light, which may thus already constitute low light for these ecotypes. *IAMH1* was upregulated in the *iamh2* mutant under low light (Figure S8A), indicating partial compensation. However, only *IAMH2* was light‐responsive in Col‐0 (Figure S8B), and this compensation did not restore hypocotyl elongation, suggesting that *IAMH2* plays a non‐redundant role in this context. Importantly, regardless of whether IAM is a minor auxin precursor or a signaling molecule, the 3′ UTR variant we identify confers a phenotypic advantage through escape from C3H14/C3H15‐mediated repression, and thus brings the C3H14/15–IAMH2 module into the context of light adaptation.

RNA‐binding proteins C3H14 and C3H15 inhibit hypocotyl elongation by recognizing a poly(U) motif in the *IAMH2* 3′ UTR (Figure 4) and likely triggering the transcript's degradation (Chen & Shyu, 1994). This mechanism exhibits significant potential for broad biological relevance. AREs are recognized as unstable mRNA signals, and their interaction with specific binding proteins is a classic model of posttranscriptional regulation (Chen & Shyu, 1994; Di Giammartino et al., 2011). Posttranscriptional regulation has proven crucial in plant shade avoidance responses; for example, the activity of the key transcription factor HY5 is finely regulated at multiple levels (Zhang et al., 2025). Our work establishes the C3H14/C3H15‐*IAMH2* module as a specific “brake” mechanism within this regulatory network (Figure 5H). Notably, beyond its role in light signaling, C3H14 is also embedded in secondary cell wall synthesis networks, being directly regulated by MYB46 (Ko et al., 2009). This dual connectivity suggests that C3H14, and potentially its homolog C3H15, may function to integrate developmental programs such as cell wall reinforcement with environmental signals like light limitation by degrading target mRNAs like *IAMH2*, potentially enabling a trade‐off between rapid elongation growth and structural integrity under fluctuating light conditions.

The phenotypic divergence in hypocotyl elongation observed between Sic and Tib ecotypes stems from a 269‐bp structural variant in the *IAMH2* 3′ UTR. This variation disrupts the conserved poly(U)‐containing single‐stranded loop in the Tib haplotype (Hap‐Tib), thereby enabling its transcript to escape C3H14/C3H15‐mediated repression (Figures 3D, E and 5A–C). This “escape mutation” carries clear adaptive significance. In high‐altitude, high‐light environments such as Tibet, *A. thaliana* seedlings face a significant risk of burial or shading under dense vegetation. Under such selective pressure, genotypes conferring rapid hypocotyl elongation would be strongly favored to enhance light capture and competitive ability. Conversely, for the Sic ecotype in low‐altitude, relatively low‐light habitats, an excessive hypocotyl elongation response may be maladaptive. This structural variant thus represents a molecular signature of local adaptation. Similar natural variation attenuating shade avoidance has been harnessed in crop breeding (Qin et al., 2023), underscoring the broad evolutionary and agricultural importance of this regulatory mechanism.

Our study delineates how a specific structural variant fine‐tunes a key adaptive trait via posttranscriptional regulation, advancing the broader paradigm that local adaptation in *A. thaliana* emerges from diverse growth strategies sculpted by natural selection. This finding aligns with established evidence that key adaptive traits, including hypocotyl elongation, are governed by complex signaling networks modulated by natural allelic variation (Atwell et al., 2010; Coluccio et al., 2011; Filiault & Maloof, 2012). A comprehensive dissection of the genetic architecture underlying these adaptations, however, necessitates a complete panorama of genomic diversity. Large‐scale resources such as the 1001 Genomes Project, while foundational, have historically depended on short‐read sequencing—an approach constrained in its ability to accurately detect and phase major insertions, deletions, and inversions (Weigel & Mott, 2009). These structural variations are now recognized as potent drivers of phenotypic divergence (He et al., 2025; Kang et al., 2023). Consequently, although our analysis encompassed 38 representative ecotypes, the absence of a comprehensive, high‐quality global SV map for *A. thaliana* may restrict a full elucidation of the genomic basis of adaptive traits like hypocotyl elongation (Kang et al., 2023). Future integration of long‐read sequencing‐based pangenome resources, such as those generated by the 1001G+ initiative (1001 Genomes Plus Consortium et al., 2024), will be indispensable to systematically uncover the contribution of noncoding regions and SVs to local adaptation, thereby furnishing a more complete genomic context for the allelic effects identified herein and in comparable studies.

## MATERIALS AND METHODS

### Plant materials and growth conditions

Seeds of the Sichuan ecotype (CS79063) and the Tibet ecotype (CS79062), together with the *iamh2* mutant (SALK_208590C; Col‐0 background), were bulk‐propagated in a controlled growth chamber (16 h 22°C light/8 h 19°C dark, 60 ± 5% RH) to minimize maternal effects. The *c3h14 c3h15* double mutant (Col‐0 background) was kindly provided by Prof. Jing‐Shi Xue (Xue et al., 2026). After surface‐sterilization (75% ethanol, 2 min; five sterile‐water rinses), seeds were sown on 0.65% agar‐solidified half‐strength Murashige & Skoog medium (Sigma, USA) containing 1% sucrose (pH 5.7). To synchronize germination, seeds were stratified in darkness at 4°C for 3 days (Col‐0 and *iamh2*) or 10 days (Sic and Tib) as previously described (Gao et al., 2025). Plates were then incubated for 48 h at 22°C under the same long‐day photoperiod used for bulking (16 h light/8 h dark, 160 ± 2 μmol m^−2^ s^−1^) before being transferred to experimental light conditions. Seedlings were subsequently cultured for 7 days under the same long‐day photoperiod (16 h light/8 h dark) at either 160 ± 2 μmol m^−2^ s^−1^ (normal light) or 7 ± 0.2 μmol m^−2^ s^−1^ (low light). Hypocotyl length was measured from the root–hypocotyl junction to the cotyledon base using ImageJ (v1.54). At least 10 seedlings per genotype and light condition were analyzed in each of three independent biological replicates.

### Genome‐wide association analysis

Hypocotyl lengths were measured by 146 recombinant inbred lines (RILs) under standardized low‐light conditions, with a minimum of 10 seedlings evaluated per line (Data S4). SV‐GWAS was conducted using the EasyOmics platform (Han et al., 2025), which implements GCTA (Yang et al., 2011) to perform association analysis with a linear mixed model (LMM) that accounts for population structure and relatedness. Phenotypic data from the RILs were integrated with genome‐wide structural variant calls in VCF format. A total of 21 341 high‐quality structural variants (minor allele frequency ≥0.05, missing rate ≤0.3, depth ≥2) were retained for analysis. Significance thresholds were determined by Bonferroni correction for multiple testing.

### Gene expression analysis

Total RNA was extracted from manually dissected hypocotyls of 9‐day‐old seedlings using TRIzol reagent (Bio Fit, USA). cDNA synthesis was performed from 1 μg of RNA using the PrimeScript RT reagent kit (Takara, Japan). Real‐time quantitative PCR (RT‐qPCR) analysis of relative gene expression was carried out with SYBR Green PCR Master Mix (Yeasen, China) utilizing a Bio‐Rad CFX96 Real‐Time System. *ACTIN2* (*At3g18780*) functioned as the internal control gene. Comprehensive primer information is presented in Data S5.

### Dual‐luciferase reporter assay

To generate reporter plasmids, the 3′ UTR sequences of *IAMH2* from the Col‐0, Sic, and Tib ecotypes were ligated downstream of the firefly luciferase (LUC) coding region in the pGreen‐0800‐LUC vector. For effector expression, the coding sequences of *C3H14* and *C3H15* were each cloned into the pBI221‐GUS vector under the control of the *CaMV35S* promoter, resulting in C‐terminal GUS translational fusions. Primer sequences employed for these molecular cloning steps are documented in Data S5.

Functional validation was conducted in *Nicotiana benthamiana* mesophyll protoplasts using a dual‐luciferase reporter system. Protoplasts were co‐transfected with the reporter and effector plasmids via PEG‐calcium‐mediated transformation. After transfection, protoplasts were maintained in darkness at 22°C for 16 h prior to lysis. Luminescence from LUC and Renilla (REN) luciferases was measured sequentially using a Dual‐Luciferase® Reporter Assay kit (Promega, USA) on a GloMax 96 microplate luminometer. The constitutively expressed REN signal served as an internal control for normalization, and relative reporter activity was calculated as the LUC/REN luminescence ratio.

### Electrophoresis mobility shift assay

Recombinant MBP‐tagged C3H14 and C3H15 proteins were heterologously produced in *E. coli* Rosetta2 (DE3) and affinity‐isolated using amylose‐resin chromatography. For binding analyses, 5′‐biotin‐labeled RNA probes derived from the *IAMH2* 3′ UTR were chemically synthesized (Sangon Biotech, China); unlabeled identical RNAs served as competitors. RNA–protein interactions were examined with a non‐radioactive EMSA kit (Viagene, USA). Binding reactions contained the purified protein and labeled probe, with competition assays incorporating a 50‐ or 100‐fold molar excess of unlabeled RNA. Complexes were resolved on native polyacrylamide gels, transferred to a nylon membrane, and visualized via chemiluminescent detection. Probe sequences are detailed in Data S5.

### Phylogenetic analysis

The *IAMH2* mRNA sequences from 38 geographically dispersed *A. thaliana* ecotypes and one *A. lyrata* outgroup were retrieved from published genomic resources (Kang et al., 2023; Nordberg et al., 2014). The *IAMH2* mRNA sequences were aligned using MEGA X software, and phylogenetic trees were constructed using the maximum likelihood method with the Hasegawa–Kishino–Yano (HKY) substitution model. Branch support was assessed by performing 1000 bootstrap replicates to evaluate the reliability of the inferred phylogenetic relationships.

### Statistics and reproducibility

Significance was assessed by unpaired two‐tailed Student's *t*‐test or one‐way ANOVA with Tukey's multiple‐comparison test (GraphPad Prism 9.0). All experiments comprised three or more biological replicates.

## AUTHOR CONTRIBUTIONS

XG, WC, and XL finished most of the analyses and experiments. XG and HL wrote the manuscript. JL revised the manuscript. YS performed parts of the bioinformatics analysis. YY, JY, and KY performed parts of the biochemical experiments.

## CONFLICT OF INTEREST

The authors declare no competing interests.

## Supporting information


**Data S1.** Annotation of candidate genes.

**Data S2.** *IAMH2* mRNA sequences from 38 *A. thaliana* ecotypes and one *A. lyrata* outgroup.

**Data S3.** Geographic and environmental data of the 38 *A. thaliana* ecotypes.

**Data S4.** Hypocotyl length of RIL population.

**Data S5.** List of primer sequences used in the study.

**Figure S1.** Solar radiation at the sampling locations and *IAMH2* expression in the Sic and Tib ecotypes.
**Figure S2**. Scheme for constructing the mapping population.
**Figure S3**. Gene structure and allelic variation of *At4g37550* (A), *At4g37540* (B), *At4g37530* (C), and *At4g37520* (D) between the Sichuan and Tibet ecotypes.
**Figure S4**. DNA sequence of *IAMH2* in the ecotypes from Col‐0, Tib, and Sic.
**Figure S5**. Identification of *iamh2* T‐DNA insertion loss‐of‐function mutants.
**Figure S6**. Functional prediction and structural analysis of the IAMH2 protein across ecotypes.
**Figure S7**. The SV in the *IAMH2* 3′ UTR contributes to variations in hypocotyl length.
**Figure S8**. Expression analysis of *IAMH1*.

## Data Availability

Elevation and solar‐radiation data were sourced from the WorldClim database (Fick & Hijmans, 2017). Using ArcMap 10.7, values for each ecotype were extracted according to its geographic coordinates (Data S3). All genetic materials are available from the corresponding authors upon request.
