# Supplementary material for: Structural variation in the IAMH2 3′ UTR underlies hypocotyl elongation during light adaptation in Arabidopsis thaliana
Source: Plant J. 2026 Oct 7;128(1):e71167. doi: 10.1111/tpj.71167 (PMC13646855; doi:10.1111/tpj.71167)
Supplement: Supplementary file 1 [file TPJ-128-0-s005.docx]

**Figure S1.** Solar radiation at the sampling locations and *IAMH2* expression in the Sic and Tib ecotypes. (A, B) Monthly (A) and annual averages (B) of daily solar radiation for the period 1970–2000. Data were sourced from the WorldClim database. The geographical coordinates of the sampling points are Sichuan (31.56402°N, 106.70624°E) and Tibet (29.68976°N, 91.1861°E). (C) RT-qPCR analysis of *IAMH2* expression in manually dissected hypocotyls from 9-day-old Sic and Tib seedlings under normal light. Data are mean ± SD (n = 3 biological replicates); significance evaluated by unpaired two-tailed Student’s *t*-test.

**Figure S2.** Scheme for constructing the mapping population. The mapping population was developed by backcrossing the F1 progeny (Sichuan × Tibet ecotypes) five times to the Tibet ecotype, using it as the recurrent parent, followed by five generations of self-pollination. Each generation was selected under a 9-day low-light induction regime for hypocotyl elongation.

**Figure S3.** Gene structure and allelic variation of *At4g37550* (A), *At4g37540* (B), *At4g37530* (C), and *At4g37520* (D) between the Sichuan and Tibet ecotypes.

**Figure S4.** DNA sequence of *IAMH2* in the ecotypes from Col-0, Tib, and Sic.

**Figure S5.** Identification of *iamh2* T-DNA insertion loss-of-function mutants. (A) Schematic of the T-DNA insertion site in SALK_208590C. (B) Genotyping of SALK_208590C by three-primer PCR. (C) RT-qPCR analysis of *IAMH2* expression in Col-0 and *iamh2* seedlings.

**Figure S6.** Functional prediction and structural analysis of the IAMH2 protein across ecotypes. (A) Amino acid sequence alignment of the IAMH2 protein from Col-0, Tib, and Sic ecotypes. (B–D) Predicted tertiary structures of IAMH2 generated by AlphaFold3. Structures derived from the Sic and Tib ecotypes are shown separately in (B) and (C), respectively, with their structural superposition presented in (D). SNP sites are represented using a stick model. (E) Comparative structural models of IAMH2 from Tib (yellow) and Sic (blue) ecotypes, predicted using the Swiss-Model server.

**Figure S7.** The SV in the *IAMH2* 3′ UTR contributes to variations in hypocotyl length. (A) Representative images of 9-day-old seedlings from different lines. Scale bar, 2 mm. (B) Quantification of hypocotyl lengths corresponding to (A). Data are mean ± SD (n = 6 seedlings); different letters indicate statistically significant differences (*P* < 0.05, one‑way ANOVA with Tukey’s multiple comparison test). (C) RT‑qPCR analysis of the *IAMH2* expression in manually dissected hypocotyls from 9‑day‑old seedlings grown under low‑light condition.

**Figure S8.** Expression analysis of *IAMH1*. (A) RT-qPCR analysis of *IAMH1* expression in manually dissected hypocotyls from 9-day-old Col-0 and *iamh2* seedlings under low light. (B) RT-qPCR analysis of *IAMH1* and *IAMH2* expression in manually dissected hypocotyls from 9-day-old Col-0 seedlings under normal or low light. Data are mean ± SD (n = 3 biological replicates); significance evaluated by unpaired two-tailed Student’s *t*-test.
